# Development of a New Colorimetric, Kinetic and Automated Ceruloplasmin Ferroxidase Activity Measurement Method

**DOI:** 10.3390/antiox11112187

**Published:** 2022-11-04

**Authors:** Salim Neşelioğlu, Esra Fırat Oğuz, Özcan Erel

**Affiliations:** 1 Department of Biochemistry, Faculty of Medicine, Yıldırım Beyazıt University, Ankara 06800, Turkey; 2 Clinical Biochemistry Laboratory, Ankara City Hospital, Ankara 06800, Turkey

**Keywords:** chromogen-free, enzyme activity, ferric iron, ferrous iron

## Abstract

Background: Ceruloplasmin plays an important role in the regulation of iron metabolism. Ceruloplasmin is an acute-phase protein known to have many metabolic effects. Its activity increases during infection, inflammation, and compensation of oxidation. In the current study, our aim is to develop a new method for the measurement of ferroxidase activity without requiring any chromogen. Methods: Venous blood samples were collected into serum separator tubes. Ferric iron ions formed by the enzyme ferroxidase were measured, both manually and fully automatically, at the 415 nm wavelength without using chromogen. These results were compared to conventional ferroxidase measurement methods and to the immunoturbidimetric ceruloplasmin measurement method. Results: The detection limit of the new assay was 14.8 U/L. The upper limit of the linearity was 1380 U/L. Precision values were calculated for high, medium, and low levels of ferroxidase activity in serum pool. The coefficient of variation was <5% for each level. Conclusion: In the present method, chromogens are not used. With its considerably low cost and short reaction time, this method is able to provide fast results, can be performed easily, and makes accurate measurements.

## 1. Introduction

Ceruloplasmin (CP) is an α2-globulin with a molecular weight of 151 kDa with six copper atoms in the structure, and is synthesized in hepatic parenchymal cells [1,2,3,4,5]. CP, one of the proteins that exhibits ferroxidase activity, plays an important role in the regulation of iron metabolism. It oxidizes ferrous iron ions to ferric iron ions, which subsequently bind to transferrin, thus preventing the formation of toxic iron products [1,5,6]. Additionally, there are two known CP homologs that also exhibit ferroxidase activity—hephaestin (HEPH) and zyklopen (HEPHL1) [7].

CP is an acute-phase protein known to have many metabolic effects. Its activity is associated with copper transport, as well as anti-inflammatory and antioxidant responses [2,3,8,9,10,11,12]. CP also plays important roles in nitric-oxide-related dilatation of the vascular endothelium [13,14,15].

Plasma CP levels have been reported to be significantly lower in protein synthesis disorders such as liver failure, malnutrition, protein-losing enteropathy, nephrotic syndrome, hereditary hypoceruloplasminemia, Wilson’s disease, Menkes disease, nutritional copper deficiency, diabetes mellitus, and glaucoma [4,16,17,18,19,20,21].

There are many CP measurement methods. One of the methods is based on the quantitative analysis of CP by measuring the oxidase activity of CP. One of the commonly adopted methods in routine analysis is the quantitative immunometric method [20,22,23]. Other methods are based on the principle of oxidation of non-physiological substrates such as o-dianizidine, p-phenylenediamine, and N,N-dimethyl-p-phenylenediamine [19,24,25,26,27]. However, the oxidase activity of CP has a higher affinity for ferrous iron ions than other substrates [28,29]. Most of the methods for the measurement of CP oxidase activity require a chromogen, which either causes the color to decrease as the substrate is utilized or causes the color to increase as the product is formed [8,23,24,27,30]. Thus, ferric iron ions are not measured directly, but rather indirectly via a chromogen. In the current study, we describe a new method for the measurement of ferroxidase activity of CP which does not require the presence of a chromogen.

## 2. Materials and Methods

### 2.1. Chemicals

Acetic acid, sodium acetate, sodium azide, ammonium iron(II) sulfate hexahydrate, p-phenylenediamine, o-dianisidine, and human ceruloplasmin were purchased from Sigma-Aldrich Chemical Co. (Milwaukee, WI, USA) and Merck Co. (Darmstadt, Germany). All chemicals were ultrapure grade, and type-I reagent-grade deionized water was used.

### 2.2. Samples

Fifty-nine venous blood samples were collected from healthy subjects after 10–12 h of fasting and placed into serum separator tubes. Samples were centrifuged at 1500× *g* for 10 min. Serum samples were aliquoted and stored at −80 °C. Informed consent of all participants has been obtained and archived. The study was approved by local ethics committee of the Yıldırım Beyazıt University Faculty of Medicine.

### 2.3. Apparatus

A Shimadzu UV-1800 spectrophotometer (Kyoto, Japan) with a temperature-controlled cuvette holder, a Cobas c501 autoanalyzer (Roche Hitachi, Mannheim, Germany), and a BNII nephelometer (Siemens, Munich, Germany) were used.

### 2.4. Colorimetric and Fully Automated Ceruloplasmin Ferroxidase Activity Measurement Method

The measurement of ferroxidase activity was performed using a colorimetric method in which the yellow products formed, ferric iron ions (Fe^3+^), were measured directly, manually, and fully automatically without using any chromogen.

The detection limit of the new method was determined by measuring the zero calibrator (a blank sample containing no analyte) ten times. Hemolysis, icterus, lipemia, and sodium azide were used for inhibition studies.

#### Assay Protocol

Sodium acetate buffer (450 mmol/L, pH: 5.8) preparation: Firstly, 450 mmol/L glacial acetic acid and 450 mmol/L sodium acetate solutions were prepared. Then, 100 mL of buffer solution with a pH value of 5.8 was obtained by mixing 6 mL of acetic acid solution and 94 mL of sodium acetate solution.

Preparation of 20 mmol/L substrate solution: 0.78 g of ammonium iron(II) sulphate hexahydrate was dissolved in 100 mL of deionized water.

The first step of the assay was the addition of 150 µL of buffer solution (450 mmol/L acetate buffer solution, pH: 5.8) to 45 μL of serum sample. After mixing, 20 μL of substrate solution (20 mmol/L iron(II) sulfate hexahydrate) was added to the reaction medium. The absorbance of yellow-colored Fe^3+^ ions, which are produced as a result of the catalytic activity of the ferroxidase enzyme, was monitored kinetically at a wavelength of 415 nm for 10 min using spectrophotometer (Table 1).

### 2.5. Ceruloplasmin Determination with Immunoturbidimetric Method

The measurement of ceruloplasmin immunoturbidimetrically is based on the principle of measuring CP using anti-ceruloplasmin-specific antiserum. The measurement was performed at 340/700 nm (primary/secondary wavelength) in this immunochemical method [31,32].

### 2.6. p-Phenylenediamine (PPD) Oxidase Activity Measurement

p-Phenylenediamine substrate solution (0.5%) and acetate buffer (pH 6.0) were used in the assay. The CP oxidase activity was measured kinetically according to the oxidation of PPD at 540 nm [11,25,33].

### 2.7. o-Dianisidine Oxidase Activity Measurement

o-Dianisidine (7.8 mmol/L) as a substrate and acetate buffer (pH 5.0) were used in the assay. The CP oxidase activity was determined via the oxidation of o-dianisidine at 460 nm [23,34,35].

### 2.8. Statistical Analysis

SPSS software Ver. 22.0 was used for statistical calculations (SPSS Inc., Chicago, IL, USA). The data were distributed normally, and independent sample t-tests were used for comparison purposes. Pearson’s correlation coefficients and linear regression analyses were performed to observe the relationships between the assays. In all analyses, a *p*-value of less than 0.05 was considered to be statistically significant.

## 3. Results

### 3.1. Optimization of Reagent 1

A sodium acetate buffer that had pH values ranging from 3.6 to 5.9 was used. Sodium acetate buffer concentration values ranged from 0 to 700 mmol/L with glacial acetic acid. The optimal pH range and sodium acetate buffer concentration were found to fall within 5.6–5.8 and 450–500 mmol/L, respectively.

### 3.2. Optimization of Reagent 2

Ammonium iron(II) sulfate hexahydrate (substrate) solutions that had concentration values ranging from 5 to 50 mmol/L were utilized. The optimal concentration was found to be 20 mmol/L.

### 3.3. Analytical Parameters

#### 3.3.1. Linearity

**Figure 1:**
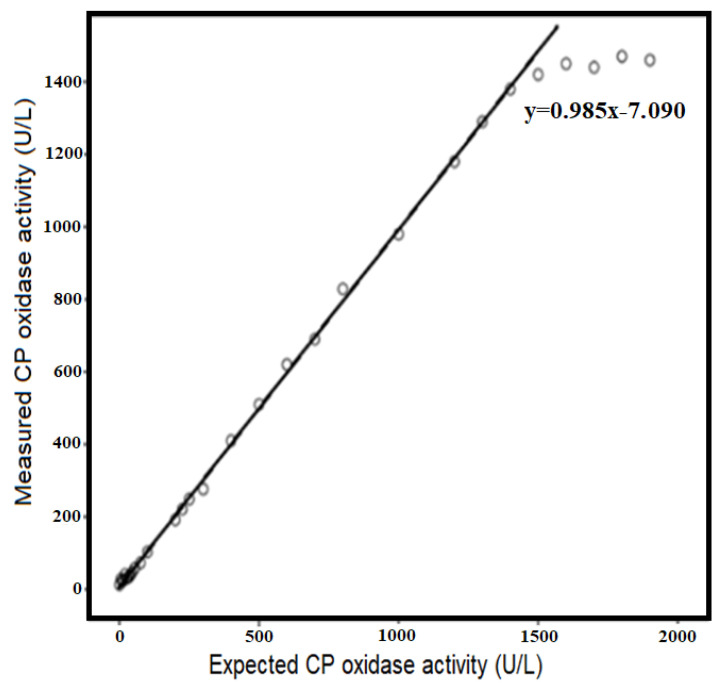
The regression line of the new method showing the linearity of CP oxidase activity using human ceruloplasmin.

Human serum CP was used to perform serial dilutions. In the assay, the upper limit of the linearity was 1380 U/L. The *r* value was 0.999 (*p* < 0.001), the slope was 0.985, and the intercept was −7.090 in the regression analyses (Figure 1).

#### 3.3.2. Detection Limit

The zero calibrator (a blank sample-containing no analyte) was measured ten times to assign the detection limit of the method. The detection limit was considered to be the mean ferroxidase activity of the zero calibrator + 3 × standard deviations, and was determined to be 14.8 U/L.

#### 3.3.3. Analytical Recovery

The recovery percentage of the newly developed method was performed by adding human ceruloplasmin to the pool of sera. The mean percentage recovery of human CP was found to be within the range 98–101%.

#### 3.3.4. Precision

Precision values were calculated for high, medium, and low levels of ferroxidase activity in serum pool. The coefficient of variation (CV %) was < 5% for each level (Table 2).

#### 3.3.5. Interference and Inhibition

There is no significant interaction with hemolysis, icterus, or lipemia. In addition, sodium azide, used as an inhibitor in inhibition studies, inhibited serum CP oxidase activity by 95–99%.

#### 3.3.6. Extinction Coefficient (ε)

The extinction coefficient was calculated according to the following formula (the Beer–Lambert law):

Enzyme activity (U/L) = μmol/min/L = (ΔAbs/time (min)) × (Vt/Vs) × 10^6^/ε × 1

ΔAbs/time, absorbance change per minute; Vt, total reaction volume; Vs, sample volume; enzyme activity; 10^6^, Conversion constant (converting between mole and micromole); l, path length.

The extinction coefficient (ε415 nm) was determined to be 3.4 × 10^2^ cm^−1^ mol^−1^ L according to the formula.

#### 3.3.7. The Reaction Kinetics and The Absorbance Spectrum

**Figure 2:**
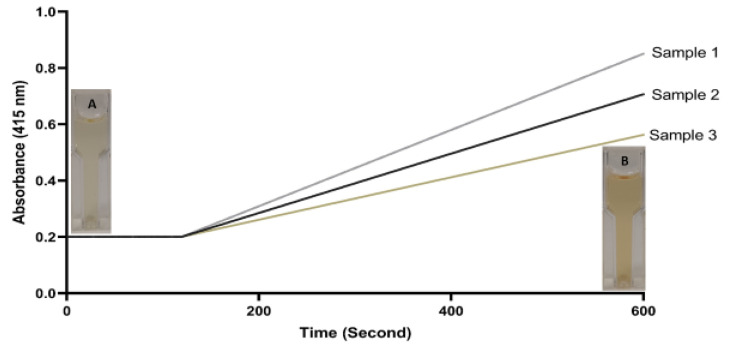
The reaction kinetics of serum samples with different activities. A (sample 2), serum + reagent 1 (buffer solution); B (sample 2), serum + reagent 1 + reagent 2 (substrate solution).

The reaction kinetics of serum samples with different activities is shown in Figure 2.

**Figure 3:**
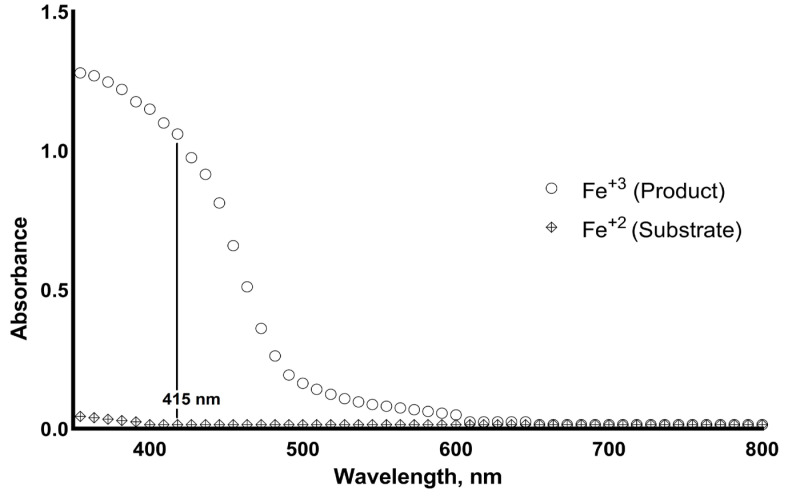
The absorbance spectrum of the substrate (Fe^+2^) and the product (Fe^+3^) formed by the ferroxidase enzyme.

The absorbance spectrum of the product (Fe^3+^) formed by the ferroxidase enzyme is shown in Figure 3.

### 3.4. Method Comparison

**Figure 4:**
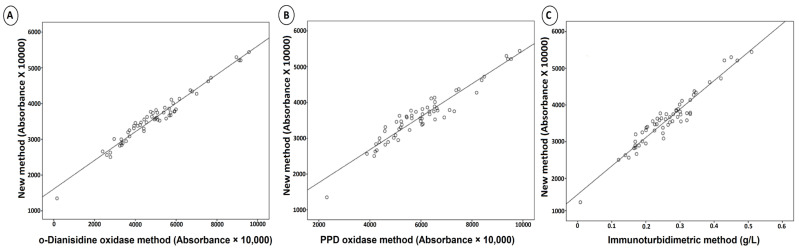
Comparison of the conventional serum ferroxidase/CP-measurement methods and the newly developed serum ferroxidase method in human blood serum. (**A**), the correlation between the new method and o-dianisidine oxidase method; (**B**), the correlation between the new method and p-phenylenediamine method; (**C**), the correlation between the new method and immunoturbidimetric method.

Serum samples with different CP oxidase concentrations were used for a comparison study. The new method showed a stronger correlation with the o-dianisidine oxidase method than with other methods. The relationship between the conventional CP measurement methods and the newly developed method is presented in Table 3 and Figure 4.

## 4. Discussion

CP is known as an acute-phase reactant, and also performs several functions involving iron metabolism, copper transport, the oxidation of some substrates, and antioxidant activity [6,7]. CP is able to oxidize various substrates such as o-dianisidine, p-phenylenediamine, and ferrous iron ions. However, the enzyme exhibits the highest level of affinity for ferrous iron ions (Fe^2+^) [28]. It has been established that CP has an important role in iron metabolism through its ferroxidase activity. CP is known to induce the release of iron from cells and to oxidize Fe^2+^ to Fe^3+^, which subsequently binds to transferrin. Unbound ferrous iron ions cause the occurrence of the Fenton reaction, which leads the formation of free radicals [7,28,36,37,38].

The principle of our method is based on the measurement of the color, formed by the conversion of Fe^2+^ ions to Fe^3+^ ions due to ferroxidase activity, at a wavelength of 415 nm. There is a need for a chromogen (such as o-dianisidine, p-phenylenediamine, or norfloxacin) in all of the enzymatic methods for the detection of the oxidase activity of ceruloplasmin. Unlike all those other methods, the originality of the method we developed is that there is no need for an additional chromogen. Conventional methods are relatively expensive because they use chromogens so that the measurement can be performed indirectly via those chromogens. However, the newly developed method is a cheaper method, and the measurement of the product (Fe^3+^) can be performed directly without the need for using any chromogen.

Similar to our method, another ferroxidase measurement method using Fe^2+^ ions as the substrate was described by Samoni BL and Ambade V. Norfloxacin was used as a chromogen for the measurement of the Fe^3+^ ions formed in this method. Notably, while the product formed in the method defined by Samoni BL and Ambade V can be measured indirectly (using a chromogen), it can be measured directly (without using chromogen) in our method [30].

In addition, PPD and o-dianisidine, the substrates in the PPD oxidase and o-dianisidine oxidase methods, are not biological substrates of the ferroxidase enzyme [28]. These methods do not use physiological substrates of the enzyme, but the results reflect the physiologically relevant oxidase activity. However, the ferrous iron ions, used as the substrate in the newly developed method, are the biological substrate of the ferroxidase enzyme, and also reflect the physiological oxidase activity. Moreover, by not using a chromogen, measurement errors that may arise from possible impurities in the chromogens or deterioration of the long-term stability of chromogens are eliminated. Therefore, we believe that the results of the new CP oxidase activity measurement method are much more reliable.

Strong correlations were obtained between the newly developed method and other conventional methods (immunoturbidimetric, p-phenylenediamine oxidase, and o-dianisidine oxidase) that do not use iron. All correlation values were greater than 95% (Table 3). In addition, strong correlation values were also observed between the immunoturbidimetric measurement method, in which the amount of CP was determined, and the enzymatic methods, in which the oxidase/ferroxidase activity of ceruloplasmin was determined (Table 3). However, a previous study indicated that some samples may have enough ferroxidase activity although the amount of ceruloplasmin is low, while other samples may have low ferroxidase activity although the amount of ceruloplasmin is high [39]. Consequently, immunoturbidimetric methods may not always reflect the function or activity of the enzyme, indicating that ceruloplasmin ferroxidase activity measurement methods are superior to immunoturbidimetric measurement methods.

The newly developed method has also high analytical performance characteristics, as well as the aforementioned originalities. Various factors have been evaluated to determine the accuracy and precision of this optimized method. Linearity (upper limit of the linearity was 1380 U/L; *r* = 0.999; *p* < 0.001), limit of detection (the detection limit was 14.8 U/L), analytical recovery (the mean percent recovery was 98–101%), and precision (the coefficient of variation/CV% was < 5 for each level) of the new method were found to be quite strong. Additionally, there are strong and linear correlation levels among enzymatic methods (*r* > 0.95; *p* < 0.001 for all comparisons). As can be seen in Table 3 and Figure 4, the strong relationship between the newly developed ferroxidase activity method and the conventional enzymatic methods (the o-dianisidine oxidase method and the p-phenylenediamine method) have been clearly demonstrated. In addition, three different serum samples with low, normal, and high ferroxidase activity were measured with the newly developed method (Figure 4). This figure shows the discrimination ability, reaction time, and absorbance levels of the method.

In the inhibition experiment with sodium azide to prove the presence of ferroxidase activity, it was revealed that the enzyme ferroxidase was inhibited at a level of 95–99%. Since the newly developed measurement method is based on kinetic monitoring, there was no significant interaction with hemolysis, icterus, or lipemia.

## 5. Conclusions

In conclusion, the newly developed kinetic method is easily applicable, can be employed with a small budget, and can be applied both automatically and manually. Moreover, it is a unique method that is different from traditional methods, since the generated product can be measured directly, without the need for chromogen use.

## Figures and Tables

**Table 1:** The procedure of the assay for fully automatic spectrophotometric devices.

Sample volume	45 μL
Reagent 1	150 μL (450 mmol/L acetate buffer solution, pH: 5.8)
Reagent 2	20 μL (20 mmol/L iron(II) sulfate hexahydrate in deionized water)
Wavelength	415 nm
Reading point	The reaction was linear with regard to the duration of the incubation period, about 10 min
Calibration type	Linear (Human CP was used as the calibrator for the assay)

**Table 2:** Intra-day and inter-day precision of the newly developed ferroxidase method.

Intra-Day Precision	Mean (*n* = 30)	Standard Deviation	CV %
High	1237	51.2	4.1
Medium	724	23.7	3.3
Low	273	13.1	4.8
Inter-day precision			
High	1293	58.6	5.5
Medium	701	27.8	4.0
Low	297	14.3	4.8

**Table 3:** The relationship between the conventional CP oxidase measurement methods and the newly developed ferroxidase method (*n* = 59).

	p-Phenylenediamine Oxidase Method	o-Dianisidine Oxidase Method	Immunoturbidimetric Method
The new method	*r* = 0.955	*r* = 0.981	*r* = 0.962
	*p* < 0.001	*p* < 0.001	*p* < 0.001
p-Phenylenediamine oxidase method		*r* = 0.976	*r* = 0.963
		*p* < 0.001	*p* < 0.001
o-Dianisidine oxidase method			*r* = 0.972
			*p* < 0.001

## Data Availability

Data is contained within the article.
